# Vitamin C supplementation in nicotine use during pregnancy: A narrative review

**DOI:** 10.1177/17455057241305265

**Published:** 2025-02-22

**Authors:** Carolin von Edlinger, Udo R Markert

**Affiliations:** Placenta Lab, Department of Obstetrics, Jena University Hospital, Jena, Germany

**Keywords:** smoking, nicotine, vitamin C, ascorbic acid, pregnancy

## Abstract

Nicotine use during pregnancy remains a widespread problem in obstetrics, leading to complications such as intrauterine growth restriction, preterm birth, stillbirth, and sudden infant death syndrome. Consistent education by medical personnel is essential, as no medication or supplement has been found to prevent the dangers of nicotine use during pregnancy. If a pregnant woman is unable to quit nicotine despite intensive efforts, vitamin C, with its antioxidant properties, may help mitigate these risks, as suggested by some studies. This review summarizes current knowledge based on publications related to vitamin C, nicotine, and pregnancy. Research was conducted on the medical literature platforms PubMed and Cochrane Library, using all relevant studies to provide a comprehensive overview of the topic. The identified studies primarily examined the impact of maternal smoking and nicotine on placental function, as well as the respiratory, cardiac, neuronal, and bone systems of the offspring. They suggest that vitamin C has a generally positive preventive or protective effect, though no study has shown complete compensation for the damage caused by nicotine. Nicotine abstinence remains the most crucial preventive measure. If this is not achievable despite intensive efforts by medical personnel, vitamin C supplementation during pregnancy may be considered. With a very low side effect profile, a daily dose of up to 500 mg can be recommended. However, further studies are necessary to provide reliable data on the effectiveness and appropriate dosage, given an ethically justifiable study approach.

## Plain language summary

This review looks at how vitamin C might protect against the harmful effects of smoking during pregnancy, based on previously published scientific papers.

Why was the study done?

Nicotine use during pregnancy poses significant risks, including restricted fetal growth, preterm birth, stillbirth, and sudden infant death syndrome. Despite efforts to educate pregnant women about these dangers, nicotine use remains common. No medication or supplement has yet been identified to effectively counteract these risks. However, there is emerging research suggesting that vitamin C, known for its antioxidant properties, could help reduce the negative impacts of nicotine use during pregnancy. This review was conducted to explore the current state of research on this topic.

What did the researchers do?

The authors reviewed existing studies that focused on the effects of maternal nicotine use and the potential protective role of vitamin C supplementation. The studies primarily examined the impact on the placenta, as well as the respiratory, cardiac, and neuronal systems of offspring exposed to maternal smoking. They sought to determine whether vitamin C could prevent or reduce the harm caused by nicotine exposure during pregnancy.

What did the researchers find?

The studies reviewed showed generally positive results, indicating that vitamin C supplementation may have a protective effect against some of the damage caused by nicotine use during pregnancy. However, none of the studies demonstrated complete compensation for the harm caused by nicotine. The researchers suggested that a daily dose of 500 mg of vitamin C during pregnancy might offer some benefit, particularly for women who feel unable to quit smoking. At this dosage, no significant side effects are expected.

What do the findings mean?

While the best way to prevent nicotine-related complications during pregnancy is still to quit smoking, vitamin C supplementation may be a helpful adjunctive measure for pregnant women who struggle to stop smoking. The findings point to the need for further research to better understand the effectiveness and appropriate dosage of vitamin C, using ethically sound study designs to explore this potential intervention.

## Introduction

Despite extensive education and advertising campaigns, nicotine use during pregnancy remains a widespread problem in obstetrics. The numbers are alarming: more than 50% of adult women who smoke continue using nicotine after becoming pregnant.^1^ Overall, this accounts for an estimated 7%–25% of pregnant women across different countries.^1^ Additionally, exposure to secondhand or passive smoke further increases this number.^1^

Nicotine, the major harmful component of smoking, reduces blood flow to the uterus and placenta, leading to hypoxia.^2^ Nicotine and its metabolite, cotinine, can easily cross the placental barrier, causing several complications for both the woman and the unborn child.^3^ These complications include an increased risk of gestational diabetes mellitus, intrauterine growth restriction, preterm birth, stillbirth, and sudden infant death syndrome (SIDS).^4^ In later childhood and adulthood, maternal smoking during pregnancy can lead to further issues such as attention deficits, depression, pulmonary deficits, hypertension, and type II diabetes mellitus in the child.^1,5–7^

Nicotine induces alterations in cholinergic transmission and DNA methylation in the unborn.^8,9^ Additionally, intracellular calcium levels and reactive oxygen species (ROS) are significantly altered in the offspring, causing cell death and inhibiting fetal growth.^10^ This occurs in a dose-dependent manner: the higher the nicotine use, the more likely the child may be harmed.^10^

Vitamin C, with its antioxidant properties, has the potential to mitigate some of the damage caused by nicotine. Ascorbic acid and dehydroascorbic acid form a redox system that buffers ROS, protecting the body from their damaging effects.^11^ The placenta separates the oxidized isoform of vitamin C from the circulating portion, and after metabolism, it crosses the placental barrier in its reduced form.^12^ Increased maternal intake of vitamin C leads to higher concentrations in the umbilical cord blood.^13^

Several studies suggest that vitamin C supplementation can have a preventive effect on the risks associated with maternal nicotine use. This narrative review provides an overview of the current state of research on this topic, focusing on the effects of vitamin C on the placenta, as well as the respiratory, cardiac, neuronal, and skeletal systems of the offspring. As most studies with strong evidence emphasize the respiratory system’s effects, our review concentrates on this area.

## Methods

A digital search of the bibliographic databases PubMed and Cochrane Library was conducted up to June 2024, using the following search terms: “pregnancy nicotine vitamin C,” “pregnancy smoking vitamin C,” “pregnancy nicotine ascorbic acid,” “pregnancy smoking ascorbic acid,” “pregnancy nicotine ascorbate,” and “pregnancy smoking ascorbate.” A total of 112 results were found. All clinical, epidemiological, and experimental studies focusing on the impact of vitamin C on maternal nicotine use during pregnancy were included in the results. Books, reviews, non-English publications, and non-peer-reviewed documents were excluded. After analyzing all results according to the mentioned criteria, only 27 studies were deemed suitable for inclusion in the results section (Table 1).

**Table 1.** Effects of vitamin C on nicotine-induced harm to offspring during pregnancy.

Study (first author, year)	Study design	Dosage of vitamin C	Effects of vitamin C supplementation on potential harm caused by nicotine
Placental function			
Abramovici, 2015^14^	RCT (*n* = 1551)	Combination of 1000 mg vitamin C and 400 IU vitamin E	Significantly protective for placental abruption and preterm birth in smokers, no effect on preeclampsia/pregnancy associated hypertension
Saylor, 2017^15^	RCT (*n* = 8)	500 mg/day	Failed to rescue site-specific pathways, circulatory processes, and histidine metabolism, but showed potential for compensatory and functional alterations
Klesges, 1998^16^	Cohort study (*n* = 1012/*n* = 613 in surface/villus study)	Dietary intake	Significant reduction on villous calcification in women of color, but not in Caucasians, protective trend for surface calcifications, not significant
Lo, 2015^17^	Study in non-human primates	50, 100, or 250 mg/kg/day	Protective effect on trophoblast cells, mitigation of reduced placental blood flow, no effect on maternal placental blood flow
Gunes, 2008^18^	Study in rats	1 mg/kg/day	No effect on lower birth weight, lower pregnancy weight gain, higher MDA and SOD in placenta
Khan, 2011^19^	Study in mice	Vitamin C: 35 mg/kg/day; vitamin E: 400 IE in 20 kg of food	No effect on nicotine induced disturbance of placenta architecture
Gallo, 2010^21^	In vitro (placental villi culture, JEG-3 as model for trophoblast cells)	5 × 10^−12^ M 5 × 10^−8^ M	Protection of placenta against deleterious effects of ROS induced by nicotine, high overdose of vitamin C induces adverse effects
Respiratory system			
McEvoy, 2014^22^	RCT (*n* = 235)	500 mg/day	Significant amelioration of pulmonary function and compliance, reduction of wheezing after the first year of life
Follow-up, 2018^23^	RCT (*n* = 225)	500 mg/day	Significant amelioration of pulmonary function of offspring at 3 and 12 months
McEvoy, 2022^24^	RCT (*n* = 192)	500 mg/day	Significant amelioration of pulmonary function of offspring persistent at the age of 5 years
Shorey-Kendrick, 2017^25^	RCT (*n* = 235; sample from McEvoy 2014^22^)	500 mg/day	Normalization of DNA methylation across multiple loci (measured in placental tissue, cord blood and buccal mucosa of infants)
McEvoy, 2017^26^ (“VCSIP”)	RCT (*n* = 222)	500 mg/day	Initial study on effects of vitamin C on nicotine-induced effects during pregnancy on airway function of offspring
Follow-up, 2019^27^	RCT (*n* = 222)	500 mg/day	Improved airway function at the age of 3 months
Follow-up, 2020^28^	RCT (*n* = 222)	500 mg/day	Significant increase in offspring’s airway function at the age of 3 and 12 months
Follow-up, 2023^29^	RCT (*n* = 212)	500 mg/day	Significantly increased airway function of offspring at the age of 5 years and significant decrease in the occurrence of wheezing
Follow-up, 2024^30^	RCT (*n* = 222)	500 mg/day	New longitudinal analysis of FEF_25–75_ at 3, 12, and 60 months of age in the offspring shows preventive effect of vitamin C on the pulmonary function
Shorey-Kendrick, 2021^31^	RCT (*n* = 96; sample from VCSIP^26^)	500 mg/day	Improvement in placental DNA methylation and gene expression associated with maternal smoking, potentially linked to better placental function and enhanced respiratory health in offspring
Shorey-Kendrick, 2024^32^	RCT (*n* = 158; sample from VCSIP^26^)	500 mg/day	Normalization of DNA methylations across multiple loci persistent in buccal mucosa and methylations associated with lung function and occurrence of wheeze at 5 years of age of offspring
Proskocil, 2005^33^	Study in rhesus monkeys	360 mg/day	Significant counteraction of reduced forced expiratory rate and surfactant apoprotein B in newborns, no effect on other endpoints such as nicotine-induced changes in body weight or chromogranin A levels
Maritz, 1993^34^	Study in rats	1 mg/kg/day	Prevention of adverse effects on neonatal lung carbohydrate, DNA and protein metabolism
Maritz, 1997^35^	Study in rats	1 mg/kg/day	No prevention of adverse effects of nicotine exposure on fetal lung development; vitamin C during lactation prevented further deterioration
Maritz, 2011^36^	Study in rats	0.5 mg/kg/day	Significant counteraction of nicotine-induced lung parenchymal injury resembling microscopic emphysema in offspring after reaching maturation
Leuchtenberger, 1984^37^	In vitro (fetal and adult lung cells)	8 mg/l	Significant increase of growth and decrease of mitotic abnormalities caused by nicotine
Other systems			
Slotkin, 2005^38^	Study in rhesus monkeys (*n* = 34)	250 mg/day	Partly protective and partly synergistic effects on brain and heart development
Slotkin, 2011^39^	Study in rhesus monkeys (*n* = 66)	50/100/250 mg/day	Prevention of nicotine-induced decrement in cardiac norepinephrine levels and increase in 5HT-5HIAA-turnover in brainstem
Naseer, 2010^42^	Study in rats	0.5 mM	Significant counteraction of nicotine-induced increase of GABA(B1) and GABA(B2)R protein expression in cortex and hippocampus
Koklu, 2006^43^	Study in rats	1 mg/kg/day	Prevents of decrease of bone lengthening caused by nicotine

ROS: reactive oxygen species; RCT: randomized controlled trial; MDA: malondialdehyde; SDO: superoxide dismutase; FEF: forced expiratory flow; VCSIP: Vitamin C to Decrease the Effects of Smoking in Pregnancy on Infant Lung Function.

## Results

### Protective effects of vitamin C in combination with vitamin E against nicotine-induced damages in pregnancy

A secondary analysis of a multicenter trial demonstrated a significant protective effect of vitamins C and E against the harmful effects of nicotine use during pregnancy. The daily intake of 1000 mg of vitamin C and 400 IU of vitamin E resulted in a reduction of placental abruptions from 1.5% to 0.1% and a decrease in preterm births from 13.9% to 10.5%. However, preeclampsia and other pregnancy-associated hypertensions were not significantly influenced by the combined intake of both vitamins.^14^

Another randomized controlled trial (RCT) examined the effect of vitamin C on post-translational histone modifications in the placental epigenome of eight women. Vitamin C failed to rescue specific pathways but showed potential for compensatory and functional alterations in placental tissue.^15^ A prospective cohort study showed a protective effect of dietary vitamin C intake against increased placental calcification due to maternal nicotine use. A significant reduction in villous calcification was observed in women of color, but not in Caucasians. There was also a slight, albeit not significant, protective effect against surface calcifications.^16^

In addition to these clinical trials, three animal studies were conducted on the topic. One experiment on non-human primates showed significant protective effects of vitamin C on placental cells and placental blood flow, while two studies on rats and mice did not demonstrate any significant effects of vitamin C on the harmful effects of nicotine.^17–19^ In the primate study, macaques exposed to nicotine via subcutaneous pumps showed that vitamin C increased the numbers of villous cytotrophoblast cells and syncytiotrophoblast growth that had been decreased by nicotine, and therefore reduced hypoxia caused by nicotine.^17, ^20^^ However, vitamin C did not significantly affect changes in maternal placental blood flow.^17^ In the rat study, markers for oxidative stress, birth weight, and pregnancy weight gain were examined, showing significant harmful effects of nicotine that vitamin C could not prevent.^18^ The mouse study, which examined passive smoke effects on the placenta, found significant disturbances in placental architecture that vitamins C and E could not prevent.^19^

An in vitro study on placental villi culture and trophoblast cells showed that vitamin C protected the placenta against ROS-induced oxidative stress from nicotine. Pretreatment with vitamin C significantly blocked the increase in malondialdehyde, a marker for oxidative stress.^21^ Additionally, vitamin C counteracted the nicotine-induced decrease in the proliferation rate of JEG-3 choriocarcinoma cells, although an high overdose of vitamin C had antiproliferative effects.^21^

Overall, clinical trials, an animal experiment on nonhuman primates, and an in vitro study show significant preventive effects of vitamin C (alone or in combination with vitamin E) against the harmful effects of nicotine during pregnancy. Two small animal studies on mice and rats showed no preventive effects but also no side effects of vitamin C. Only the in vitro study showed adverse effects with high overdose exposure, unlikely to be reached via supplementation.

### Effects of nicotine and vitamin C on the respiratory system of offspring

Several studies have focused on the respiratory system of offspring from women using nicotine during pregnancy. Two large RCTs with follow-up studies have been performed addressing potential vitamin C prevention of respiratory system damage in offspring. The first major study in 2014 tested the respiratory function of offspring using forced expiratory flow metrics (FEF_25_, FEF_25–75_, FEF_50_, FEF_75_) and FEV1 in 235 women who received either placebo or 500 mg of vitamin C per day during pregnancy.^22^ Nicotine significantly harmed respiratory function and induced increased wheezing in the offspring, while vitamin C showed a significant preventive effect, persisting at 3 and 12 months of age.^22,23^ A follow-up study in 2022 demonstrated continued preventive effects at 5 years of age.^24^

Another study, using the same group of women, focused on the changes in DNA methylation caused by nicotine use during pregnancy and the effects of concurrent vitamin C use.^25^ Changes in multiple loci were explored in placental tissue, cord blood, and the infant’s buccal mucosa as a surrogate for airway epithelium. Nicotine use during pregnancy showed characteristic changes in all tissue types, while concurrent vitamin C use induced normalization in DNA methylation across multiple loci in all tissues.^25^ Specifically, in placental tissue, 267 hypomethylations and 191 hypermethylations caused by nicotine were restored by 64.42% and 51.31%, respectively, with vitamin C treatment. In cord blood, 300 hypomethylations and 209 hypermethylations were restored by 81.33% and 59.33%.^25^ In buccal cells, 214 hypomethylations and 227 hypermethylations were restored by 87.85% and 64.32%. Significant enrichment of restored CpGs was associated with improved pulmonary function at birth in all three tissues, while hypomethylated CpGs reversed by vitamin C treatment in cord blood were linked to reduced wheezing at 1 year of age.^25^

The second large RCT (“Vitamin C to Decrease the Effects of Smoking in Pregnancy on Infant Lung Function”-study [VCSIP] trial) was performed on 222 women to investigate the preventive effect of 500 mg of vitamin C on the harmful effects of nicotine use during pregnancy on the respiratory system of the offspring.^26^ The study measured the forced expiratory flow, specifically FEF_75_. At the age of 3 months, the airway function of the offspring improved significantly.^27^ A preventive effect of vitamin C on the respiratory system was also demonstrated at 12 months and 5 years.^28,29^ The higher incidence of wheezing at the age of 5 years after maternal nicotine use during pregnancy was significantly reduced through concurrent application of vitamin C.^29^ A new longitudinal analysis of FEF_25–75_ at 3, 12, and 60 months of age in the offspring further confirmed the preventive effect of vitamin C.^30^

Data from this study were used for a new analysis of smoking-induced changes in DNA methylation and the effects of concurrent vitamin C use.^31,32^ Vitamin C significantly prevented DNA methylation changes and gene expression in pathways potentially linked to improved placental function and offspring respiratory health.^31^ Additionally, a pattern of normalization in DNA methylation by vitamin C supplementation across multiple loci persisted in the buccal mucosa of infants at 5 years of age. These methylations were associated with lung function and the occurrence of wheezing at 5 years of age.^32^

All reviewed RCTs are based on a highly reliable study design and show preventive effects on the respiratory system of the offspring. Several studies are follow-ups, including the same group of women. A limiting factor of the VCSIP trial is the starting point of vitamin C supplementation at 23 weeks of gestation, which was due to a knowledge gap regarding the ideal initiation time. An earlier start could have shown a higher prevention of the harmful effects of nicotine during pregnancy.^26^

Apart from these RCTs, few animal experiments have been conducted on this topic. A study on pregnant rhesus monkeys, randomized to nicotine (2 mg/kg/day) or nicotine plus 250 mg of vitamin C daily, showed that nicotine significantly reduces forced expiratory flow and increases surfactant apoprotein B in newborns.^33^ Both effects were significantly counteracted by vitamin C. Other endpoints, such as nicotine-induced changes in body weight or chromogranin A, were not affected by vitamin C.^33^ Three studies examined the effect of nicotine and vitamin C in rats. One study reported a significant preventive effect of vitamin C supplementation during pregnancy and lactation on nicotine-induced changes in carbohydrate, DNA, and protein metabolism.^34^ The same group found no significant improvement from vitamin C supplementation during pregnancy on the harmful effects of nicotine on several lung parameters, such as the radial alveolar count or the linear intercept.^35^ However, further deterioration was prevented by vitamin C supplementation during lactation.^35^ Another study observed a significant reduction in nicotine-induced lung tissue damage, resembling microscopic emphysema, in mature rats whose mothers were supplemented with 0.5 mg/kg of vitamin C during pregnancy and lactation alongside nicotine exposure.^36^

One in vitro study has demonstrated that vitamin C significantly stimulates growth and reduces mitotic abnormalities caused by nicotine in fetal and adult lung cells. This study hypothesizes that vitamin C could potentially prevent the development of nicotine-induced lung cancer; however, these results are solely based on this single in vitro study.^37^

Overall, all clinical studies, most animal studies, and the in vitro study demonstrate a preventive effect of vitamin C on the harmful effects to the respiratory system of offspring caused by nicotine use during pregnancy. However, due to the limited number of RCTs, more research, especially high-evidence RCTs, is needed to reinforce the current state of knowledge.

### Effects of nicotine and vitamin C on other organ systems

Few studies have examined the impact of vitamin C on the harmful effects of nicotine on organ systems other than the placenta and respiratory system. For this review, we found four studies, including two in non-human primates and two in rats.

Effects on the neuronal and cardiac systems have been studied in rhesus monkeys. They were continuously infused with either bacteriostatic water or 2 mg/kg/day nicotine via osmotic minipumps, achieving serum levels similar to those in smoking pregnant women.^38–40^ The vitamin C group received 250 mg of vitamin C daily via chewable tablets. After cesarean section, tissue samples of fetal heart, lung, and brain were dissected. Nicotine caused multiple changes in neuronal cholinergic function, cell signaling, and cell damage in the heart, lung, and brain. Vitamin C protected against some nicotine-induced neurotoxic and peripheral damage, though it unexpectedly worsened certain aspects.^38^ It enhanced the nicotine-induced upregulation of different cholinergic receptors in some brain regions, such as the frontal cortex, while it significantly counteracted changes in the total protein/DNA ratio in the frontal cortex and completely protected the caudate from reduced DNA concentration with a corresponding rise in the total protein/DNA ratio. Additionally, vitamin C prevented the nicotine-induced decline in cardiac βARs but worsened the decline in βARs in fetal lung tissue.^38^ Overall, the reasons for these differing effects are unclear. Limitations include the study model, the small number of examined animals, and the continuous infusion of nicotine, which differs from the intermittent exposure typically seen with cigarette smoking.^38^

The effects of vitamin C on nicotine-induced brain damage have also been reported. In this study, rhesus monkeys received nicotine or placebo, with or without vitamin C, using the same study design as the previous study.^38^ Two major abnormalities in the monoamine pathway known to be affected in SIDS were examined: the brainstem serotonergic pathways and cardiac sympathetic innervations.^39,41^ Nicotine induced generalized hyperactivity in both pathways, while the coadministration of vitamin C significantly blocked some of these effects. Vitamin C did not prevent the nicotine-induced elevation of brainstem 5-HT and 5-HIAA, but it significantly prevented the increase in their turnover and subsequent hyperactivity. Furthermore, vitamin C completely prevented the decrement in cardiac norepinephrine levels caused by nicotine.^39^

In rats, nicotine exposure increased GABA(B1) and GABA(B2) receptor protein expression in the cortex and hippocampus, which vitamin C significantly counteracted.^42^ Another study found that nicotine exposure during pregnancy and lactation resulted in decreased bone lengthening in offspring, which vitamin C prevented.^43^

## Discussion

Smoking during pregnancy continues to be a widespread problem worldwide.^1^ Several damaging effects are caused by its major metabolite, nicotine.^2^ Vitamin C, with its antioxidant capacity, shows several preventive effects on damages caused by nicotine use during pregnancy to the placenta and the offspring. High dietary intake of vitamin C induces a significant reduction in nicotine-induced villus calcification, while 500 mg daily has shown potential for compensatory and functional alterations in clinical studies.^15,16^ A RCT demonstrated a significant prevention of placental abruption and preterm birth in smokers with high-dose vitamin C combined with vitamin E.^14^ Several animal and in vitro studies have described different preventive effects of vitamin C, but not every endpoint was significantly influenced. None of these studies reported side effects.^17–19,21^

Preventive effects of vitamin C on nicotine-induced respiratory system damage in offspring have been described in two large RCTs with follow-up studies showing significant amelioration of pulmonary function disorders up to 5 years of age.^22–24,26–29^ Nicotine-induced DNA methylation and gene expression changes linked to respiratory health were significantly counteracted by vitamin C in follow-up studies.^25,31,32^ Animal studies also showed protective effects of vitamin C nicotine-induced damage like a counteraction of reduced forced expiratory rate, surfactant apoprotein B or prevention of adverse effects on carbohydrate, DNA, and protein metabolism of the neonate’s lung.^33–36^ In vitro an anticancerogenic effect on fetal lung cells has been shown.^37^ Preventive effects of vitamin C coadministration during pregnancy have been demonstrated also on neural GABA channels and bone lengthening damaged by nicotine.^42^ Non-human primate studies suggested preventive effects on SIDS and brain and heart development disorders caused by nicotine use during pregnancy.^39^

## Conclusion

The findings suggest that vitamin C supplementation has the potential to reduce the harmful effects of nicotine on the offspring. Food supplements or medications that mitigate nicotine-induced effects are crucial for global health, alongside robust anti-smoking campaigns. Since vitamin C is both inexpensive and readily accessible, its coadministration could be a practical way to reduce the risk of damage in addition to efforts by medical personnel to promote smoking cessation. A dosage of 500 mg/day, as used in large RCTs, is well tolerated and sufficient to saturate vitamin C receptors.^44^ No side effects at this dosage have been reported.^45^ Pregnant smokers taking multivitamin preparations should be aware that their vitamin C levels might still be insufficient.^46^

However, taking vitamin C cannot replace abstaining from nicotine during pregnancy. The protective effect is significant for specific damages but does not offer complete compensation. The relatively small number of studies in this field highlights the need for further research to confirm findings and optimize dosage and timing to counteract nicotine’s harmful effects on pregnant women and their offspring.

## Declarations

**Ethics approval and consent to participate:** Not applicable, as the study is based exclusively on published literature.

**Consent for publication:** Not applicable.

**Availability of data and materials:** Not applicable.
